# Response of soil respiration to changes in soil temperature and water table level in drained and restored peatlands of the southeastern United States

**DOI:** 10.1186/s13021-022-00219-5

**Published:** 2022-11-19

**Authors:** E. E. Swails, M. Ardón, K. W. Krauss, A. L. Peralta, R. E. Emanuel, A. M. Helton, J. L. Morse, L. Gutenberg, N. Cormier, D. Shoch, S. Settlemyer, E. Soderholm, B. P. Boutin, C. Peoples, S. Ward

**Affiliations:** 1 TerraCarbon LLC, Peoria, IL USA; 2 North Carolina State University, Raleigh, NC USA; 3 U.S. Geological Survey, Wetland and Aquatic Research Center, Lafayette, LA USA; 4 East Carolina University, Greenville, NC USA; 5 Duke University, Durham, NC USA; 6 University of Connecticut, Storrs, CT USA; 7 Portland State University, Portland, OR USA; 8 George Mason University, Fairfax, VA USA; 9 School of Natural Sciences, Macquarie University, Sydney, NSW Australia; 10 The Nature Conservancy, North Carolina Chapter, Durham, NC USA; 11 United States Fish and Wildlife Service, Raleigh Ecological Services Field Office, Raleigh, NC USA; 12 Center for International Forestry Research, Bogor, Indonesia

**Keywords:** Land-use change, GHG emissions, Pocosin, Carbon dioxide, Drainage, Hydrological restoration, Climate change

## Abstract

**Background:**

Extensive drainage of peatlands in the southeastern United States coastal plain for the purposes of agriculture and timber harvesting has led to large releases of soil carbon as carbon dioxide (CO_2_) due to enhanced peat decomposition. Growth in mechanisms that provide financial incentives for reducing emissions from land use and land-use change could increase funding for hydrological restoration that reduces peat CO_2_ emissions from these ecosystems. Measuring soil respiration and physical drivers across a range of site characteristics and land use histories is valuable for understanding how CO_2_ emissions from peat decomposition may respond to raising water table levels. We combined measurements of total soil respiration, depth to water table from soil surface, and soil temperature from drained and restored peatlands at three locations in eastern North Carolina and one location in southeastern Virginia to investigate relationships among total soil respiration and physical drivers, and to develop models relating total soil respiration to parameters that can be easily measured and monitored in the field.

**Results:**

Total soil respiration increased with deeper water tables and warmer soil temperatures in both drained and hydrologically restored peatlands. Variation in soil respiration was more strongly linked to soil temperature at drained (R^2^ = 0.57, p < 0.0001) than restored sites (R^2^ = 0.28, p < 0.0001).

**Conclusions:**

The results suggest that drainage amplifies the impact of warming temperatures on peat decomposition. Proxy measurements for estimation of CO_2_ emissions from peat decomposition represent a considerable cost reduction compared to direct soil flux measurements for land managers contemplating the potential climate impact of restoring drained peatland sites. Research can help to increase understanding of factors influencing variation in soil respiration in addition to physical variables such as depth to water table and soil temperature.

**Supplementary Information:**

The online version contains supplementary material available at 10.1186/s13021-022-00219-5.

## Background

Peatlands cover less than 3% of land area [1] but account for 25% of soil carbon storage [2], thereby playing a disproportionately important role in the global carbon cycle. Intact peatlands are seasonally or permanently water-logged ecosystems where vegetation litter input exceeds soil organic matter (SOM) decomposition, leading to the accumulation of carbon-rich peat deposits. Conversion and drainage of peatlands alters C inputs to peat from vegetation [3] and accelerates aerobic peat decomposition by enhancing oxygen availability, thereby increasing peat CO_2_ emissions (4–6). As a result of intensifying anthropogenic disturbance, peatlands have become a growing source of greenhouse gas (GHG) emissions to the atmosphere [7–9] with drained peatlands accounting for an estimated 3% of global anthropogenic CO_2_ emissions [10, 11].

Prior to widespread conversion in the second half of the twentieth century, forested peatlands covered over 1.5 million hectares of the southeastern United States (U.S.) coastal plain from Virginia to northern Florida [12]. Most of these peatlands have been drained and converted for agriculture and timber production [13, 14], with roughly half of this conversion occurring prior to the 1980s [14, 15]. Consequently, peatland soils in the southeastern United States are a major source of anthropogenically driven CO_2_ emissions [16]. Recently, there has been interest in hydrological restoration of drained peatlands in the southeastern United States as a means to reduce anthropogenic GHG emissions and support climate change mitigation [17].

Peatland hydrological restoration is achieved through improving water management capabilities and altering local water table levels to mimic pre-drainage conditions. Water control structures can be installed within ditches to capture and hold rainfall, slowing drainage, and re-wetting the drained peat. Raising the water table level in drained peatlands in the southeastern U.S. coastal plain has been found to reduce CO_2_ fluxes from soils [18, 19] without always contributing to large concomitant increases in CH_4_ [20], as is the case in other regions (e.g., 21–23). Globally, peatland water-table drawdown attributed to rising temperatures and anthropogenic activities has a net warming effect on the climate due to increased CO_2_ emissions that offset CH_4_ emission reductions [24]. Restoration of peatlands converted to cropland and pasture in Virginia, North Carolina, and South Carolina could reduce up to 1.1–1.5 Tg CO_2_ emissions over the next decade by decreasing rates of peat SOM decomposition [16]. Peatland hydrological restoration generates numerous additional benefits, including reducing risk of wildfires and their associated negative impacts on human populations [25, 26] and increasing habitat for native wildlife [27]. Restoration also improves regional water quality [28], helps to protect downstream estuarine habitats [29], and controls flooding offsite [30].

Despite the broad benefits, restoration has been limited and large areas of drained peatlands remain [16]. Mechanisms that provide financial incentives for reducing emissions from land use and land-use change (e.g. REDD +) offer options for land managers to fund conservation of historically wet peatlands as well as hydrologic restoration [17]. In order for these mechanisms to succeed, accurate estimates of GHG emission reductions are needed, and the development of practical estimation methods are being pursued. For example, the American Carbon Registry has approved a carbon offset methodology that establishes standardized procedures to monitor and account for the GHG benefits associated with restoring drained peatlands in the southeastern U.S. coastal plain, offering the possibility to credit reductions in CO_2_ emissions from peat decomposition modeled as a function of one or more proxy variables [31].

Depth to water table has been considered the dominant biogeophysical control on peat decomposition, but field observations are not consistent [5, 32, 33]. Multiple factors in addition to water table level control soil respiration in peatlands such as soil temperature [34, 35], peat chemistry [33, 34, 36], and vegetation [35, 37]. In ex situ experiments in southeastern U.S. peatlands, increased soil temperature causes an exponential increase in microbial respiration over a large temperature range [34] while SOM phenolic content acts as a control on peat decomposition rate [33, 37]. Soil respiration also varies with vegetation structure and composition [38, 39]. Therefore, sampling soil respiration and physical drivers across a range of climatic conditions, peat characteristics, vegetation, and land-use histories is valuable for understanding how CO_2_ emissions from peat decomposition in restored peatlands may respond to changes in easily measurable physical parameters such as depth to water table and soil temperature.

We compiled measurements of total soil respiration (combined root respiration and heterotrophic respiration from peat decomposition), water table level, and soil temperature from drained and restored peatlands at three locations in eastern North Carolina and one location in southeastern Virginia, to investigate relationships among soil respiration and physical drivers across a range of site characteristics and land-use histories, including drained and restored sites. We ask the following questions about the relationships among soil respiration and physical drivers in peatlands of the southeastern U.S. coastal plain: (1) Can water table level and soil temperature explain variation in soil respiration in drained and restored peatlands?, and (2) Do relationships among soil respiration, water table level, and soil temperature differ according to peatland drainage status? In this study, we focused on soil respiration as it is one of the main components of the peat C budget [4, 40].

## Methods

### Site descriptions

**Fig. 1:**
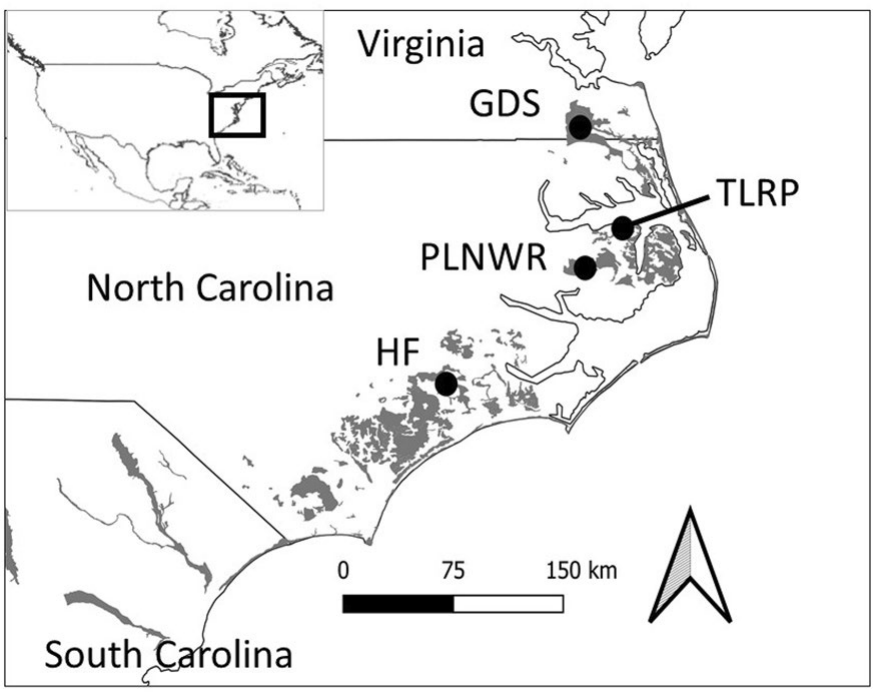
Geographic locations of peatland study sites in eastern North Carolina and southeastern Virginia. Boxed area within inset shows region within the United States. Study site locations are marked by circles. Peatlands are indicated by grey areas (Source: 1). GDS: Great Dismal Swamp National Wildlife Refuge; PLNWR: Pocosin Lakes National Wildlife Refuge; TLRP: Great Dismal Swamp Mitigation Bank Timberlake Restoration Project; HF: Hofmann Forest

Study sites were located on drained and restored peatlands in eastern North Carolina and southeastern Virginia (Fig. 1). Peatland soils in the region typically range from 1 to 3 m in depth [12]. A total of 822 observations of total soil respiration previously collected from 77 plots at 10 study sites located within Great Dismal Swamp National Wildlife Refuge (GDS) [35], Pocosin Lakes National Wildlife Refuge (PLNWR) [18, 33], Great Dismal Swamp Mitigation Bank Timberlake Restoration Project (TLRP) [19], and North Carolina State University Hofmann Forest (HF) [41] were included in our analysis (Table 1). Peatland sites included in our study represented a range of peat characteristics (Table 2). Land-use history and land management practices at each location are described in the Supplementary Information (Additional file 1). Site selection was based on availability of original data for analysis in this study and for consistency in data collection methods across sites. At each site, measurements were collected once every month to two months over partially overlapping study periods spanning eleven years (2007–2017). In all studies, total soil respiration was measured as soil-to-atmosphere CO_2_ flux from in situ dynamic or static, opaque chambers. Dynamic chambers were used at GDS (Los Gatos Research Ultra-Portable Greenhouse Gas Analyzer, San Jose, California; 35) and HF (EGM-4, SRC-1, PP Systems International, Inc., Amesbury, Massachusetts, USA; 41). At TLRP gas samples were collected from static chambers and analyzed on a Shimadzu 17A gas chromatograph [19]. At PLNWR CO_2_ fluxes were measured with a portable infrared gas analyzer (LiCor–6400–XT, Nebraska, USA; 33) from 2011 to 2013 and from 2016 to 2017 gas samples collected from static chambers were analyzed using a GC2014 Shimadzu gas chromatograph [18]. At all sites three to four replicate chambers were installed at each plot. Chamber placement excluded large trees and shrubs, and any herbaceous vegetation within chambers was clipped to the ground prior to measurement of CO_2_ flux. Therefore, CO_2_ flux measurements do not include plant uptake and should be interpreted as total soil respiration. Measurements of soil temperature and water table level were collected nearby at the same time as measurements of soil respiration. At TLRP soil temperature was measured at 5 cm and at all other locations at 10 cm. Water table level measurements were ordinarily collected from wells adjacent to chambers, but at GDS water table level measurements were obtained from continuously monitored U.S. Geological Survey (USGS) groundwater wells installed at sampling plots.

**Table 1:** Location, measurement period and mean annual precipitation (mm), mean maximum (Max T_A_) and minimum (Min T_A_) daily air temperature (^o^C) during the measurement period, drainage status, dominant vegetation, number of plots and observations (n), and associated study at peatland sites in eastern North Carolina and southeastern Virginia

Location	Site	Period	Precipitation	Max T_A_	Min T_A_	Status	Vegetation	Plots	n	Study
GDS	G-D-M	2015–2017	1364 ± 103	23.0 ± 0.2	11.1 ± 0.2	Drained	Maple-gum	3	63	35
	G-D-P					Drained	Pine-shrub	3	56	
	G-D-C					Drained	Cedar	3	34	
HF	H–D	2011–2012	1137 ± 128	23.6 ± 0.0	11.9 ± 0.2	Drained	Herbaceous	3	68	41
	H-R					Restored	Pine	3	87	
PLNWR	P-D-1	2011–2013	1364 ± 75	21.3 ± 0.4	10.0 ± 0.3	Drained	Herbaceous	3	40	USFWS, reported in 33
	P-R-1					Restored	Shrub	3	48	
	P-D-2	2016–2017	1743 ± 245	22.6 ± 0.1	11.6 ± 0.1	Drained	Herbaceous	2	26	18
	P-R-2					Restored	Shrub	2	26	
TLRP	T-R	2007–2009	1151 ± 189	22.9 ± 0.3	10.9 ± 0.0	Restored	Cypress-oak	33	293	19
		2011–2012	1352 ± 112	21.8 ± 0.1	10.2 ± 0.3			19	81	Helton, unpublished data

Values are mean ± standard error. Annual precipitation and daily maximum and minimum air temperatures were obtained from U.S. National Oceanic and Atmospheric Administration.

*GDS* great dismal swamp national wildlife refuge, *HF* North Carolina State University Hofmann Forest, *PLNWR* pocosin lakes national wildlife refuge, *TLRP* great dismal swamp mitigation bank timberlake restoration project

**Table 2:** Peat bulk density (BD), total carbon (C) content (%) and carbon to nitrogen ratio (CN) at peatland locations in eastern North Carolina and southeastern Virginia

Location	BD	C	CN	Source
GDS	0.2 ± 0.0	52.5 ± 1.1	44.6 ± 3.3	42
HF	0.8 ± 0.1	–	–	41
PLNWR	–	53.1 ± 0.8	44.3 ± 0.8	33
TLRP	0.7 ± 0.1	17.5 ± 2.4	25.0 ± 0.7	19

*GDS* great dismal swamp national wildlife refuge, *HF* North Carolina State University Hofmann Forest, *PLNWR* pocosin lakes national wildlife refuge, *TLRP* great dismal swamp mitigation bank timberlake restoration project

At PLNWR, during the study period from April 2016 to October 2017, hydrological conditions at one site were restored in March 2017 (P-R-2), decreasing depth to water table by 65% compared to pre-restoration conditions [18]. One additional site at PLNWR was restored circa 1990 (P-R-1). At TLRP and HF restored sites water tables were raised to mimic pre-drainage levels in 2004 and 2005, respectively.

### Statistical analysis

Annual total soil respiration, depth to water table, and soil respiration were calculated for each plot using linear interpolation between measurement dates. Mean annual values were computed for multi-year studies. Site-level means were calculated by averaging the plot-level means. We used the Kruskal–Wallis test to compare mean annual total soil respiration, depth to water table, and soil temperature among locations in drained (GDS, HF, PLNWR) and restored (HF, PLNWR, TLRP) peatlands. At locations with more than one site, the average of site-level values was calculated and error was propagated using the Gaussian error propagation method. We used the Wilcoxon Rank Sum test to compare total soil respiration, depth to water table, and soil temperature in drained and restored peatlands using the mean annual site-level values (n = 6 and n = 4 for drained and restored peatlands, respectively).

To test for relationships among total soil respiration, water table level, and soil temperature within and across drained and restored sites we used simple regression using the monthly observations at each plot. We used multiple regression to investigate the combined influence of water table level and soil temperature on soil respiration across drained and restored sites. The response variable, total soil respiration, was transformed to meet normality and homoscedastic variance assumptions of ordinary least squares regression [43]. Since we had no a priori reason for selecting a specific transformation, we used the Box-Cox procedure for estimating the best transformation [44]. The result (λ = 0.25) is equivalent to the quadratic root transformation. This type of transformation is useful when the variance of the dependent variable is not independent of the mean [43] as was the case with our data.

The datasets from the four geographic locations included observations where only water table level (n = 709) or only soil temperature (n = 693) was measured concurrently with total soil respiration, as well as observations where both water table level and soil temperature were measured at the same time as total soil respiration (n = 583). For multiple linear regression, we used only observations where both water table level and soil temperature were measured concurrently with soil respiration. We selected a subset (n = 10) of concurrent measurements of soil respiration, water table level, and soil temperature at P-R-2 to withhold from regression analysis to test the univariate and multiple regression models. The subset was selected to cover the range of typical climatic conditions over a calendar year. Therefore, the models relating total soil respiration to water table level, to soil temperature, and to the combined influence of water table level and soil respiration, were trained with 699, 683, and 573 observations, respectively.

We used mixed-design Analysis of Covariance (ANCOVA) to investigate potential effects of drainage status and location on the relationships among total soil respiration, water table level, and soil temperature [43]. We treated location (TLRP, HF, PLNWR, GDS) as a random effect nested within drainage status (drained, restored). All statistical analyses were computed using R Statistical Software (v4.2.0; 45). We set α equal to 0.05 for all tests of significance.

## Results

### Variation in total soil respiration, depth to water table, and soil temperature

Total soil respiration rates measured over the study periods at the four locations ranged from 0.6 mg CO_2_ m^−2^ h^−1^ (TLRP, Apr 2009) to 2.4 g CO_2_ m^−2^ h^−1^ (HF, Jul 2012). Depth to water table ranged from 212.6 cm below the soil surface (GDS, Sep 2015) to 57 cm above the soil surface (TLRP, Jun 2009) while soil temperature ranged from 3.9 °C (TLRP, Jan 2008) to 42.2 °C (HF, Jul 2011). The extremely high soil temperature measurement in a drained, deforested peatland at HF in July 2011 coincided with a historic heat wave in the continental United States [46].

Table 3 presents mean annual total soil respiration, depth to water table, and soil temperature at each site contributing data to model development as well as mean values in drained and restored peatlands at each location. Mean annual total soil respiration ranged from 20.8 Mg CO_2_ ha^−1^ yr^−1^ (P-R-1 and TLRP) to 71.2 Mg CO_2_ ha^−1^ yr^−1^ (H-R). At the restored forested site at HF (H-R) soil respiration was approximately three times greater than soil respiration at restored other sites, and it tended to be greater than the drained site with herbaceous vegetation cover at the same location (Table 3). Mean annual depth to water table ranged from 86.2 cm (GDS3) to 7.9 cm (TLRP). Mean annual depth to water table was significantly less at TLRP compared to restored sites at HF and PLNWR (Table 3). Mean annual soil temperature ranged from 15.0 °C (G-D-P) to 22.9 °C (P-R-2). Soil temperature measured at TLRP fell within this range, indicating that the impact of differences in measurement depth on soil temperature was negligible (Table 3). Soil temperature at HF was significantly higher at HF drained site compared to other drained sites.

**Table 3:** Mean annual total soil respiration (Mg CO_2_ ha^−1^ yr^−1^), depth to water table (cm), and soil temperature (^o^C) measured in peatland study sites in eastern North Carolina and southeastern Virginia

Location	Site	Land use	Total soil respiration	Water table depth	Soil temperature
GDS		Drained	25.9 ± 7.4^a^ (3)	75.5 ± 85.1 (3)	15.4 ± 1.6^a^ (3)
GD1	G-D-M	Drained	24.3 ± 3.3 (3)	76.4 ± 28.9 (3)	15.6 ± 0.5 (3)
GD2	G-D-P	Drained	27.5 ± 6.2 (3)	63.8 ± 66.0 (3)	15.0 ± 1.5 (3)
GD3	G-D-C	Drained	25.8 ± 2.4 (3)	86.2 ± 45.2 (3)	15.5 ± 0.2 (3)
HF		Drained	54.1 ± 10.8^b^ (3)	44.4 ± 8.9 (3)	20.8 ± 0.6^b^ (3)
PLNWR		Drained	31.5 ± 23.7^a^ (2)	62.7 ± 12.4 (2)	18.1 ± − (1)
	P-D-1	Drained	26.3 ± 4.9 (3)	80.3 ± 7.8 (3)	–
	P-D-2	Drained	36.7 ± 23.2 (4)	45.0 ± 9.6 (4)	18.1 ± 2.4 (4)
HF		Restored	71.2 ± 9.1^α^ (3)	38.3 ± 8.9^α^ (3)	18.0 ± 0.3 (3)
PLNWR		Restored	24.6 ± 4.6^β^ (2)	36.1 ± 22.1^α^ (2)	22.9 ± − (1)
	P-R-1	Restored	20.8 ± 2.5 (3)	47.8 ± 22.0 (3)	–
	P-R-2	Restored	28.4 ± 3.9 (2)	24.3 ± 2.0 (2)	22.9 ± 0.4 (2)
TLRP		Restored	20.8 ± 2.5^β^ (33)	7.9 ± 16.6^β^ (33)	18.9 ± 1.6 (33)

Values are mean ± standard error (number of plots included) calculated by averaging plot-level values at each site.

*GDS* great dismal swamp national wildlife refuge, *HF* North Carolina State University Hofmann Forest, *PLNWR* pocosin lakes national wildlife refuge, *TLRP* great dismal swamp mitigation bank timberlake restoration project.

Significant differences among locations are indicated by a, b for drained peatlands and by α and β for restored peatlands. No letters are displayed in the absence of a significant difference among locations

Mean annual depth to water table was greater in drained (66.0 ± 7.4 cm) than restored plots (29.6 ± 18.7 cm) (p = 0.04) but drainage status did not have a significant effect on total soil respiration or soil temperature (Table 4).

**Table 4:** Mean annual total soil respiration (Mg CO_2_ ha^−1^ yr^−1^), depth to water table (cm), and soil temperature (^o^C) in drained and restored peatlands in the southeastern United States

Land use	Total soil respiration	Depth to water table	Soil temperature
Drained	32.5 ± 4.7 (6)	66.0 ± 7.4^a^ (6)	17.0 ± 2.5 (5)
Restored	35.3 ± 15.3 (4)	29.6 ± 8.7^b^ (4)	19.9 ± 1.5 (3)

Values are mean ± standard error (number of sites included) calculated by averaging site-level means.

Significant differences between drained and restored peatlands are indicated by a, b. No letters are displayed in the absence of a significant difference

### Relationships between total soil respiration and environmental drivers

**Fig. 2:**
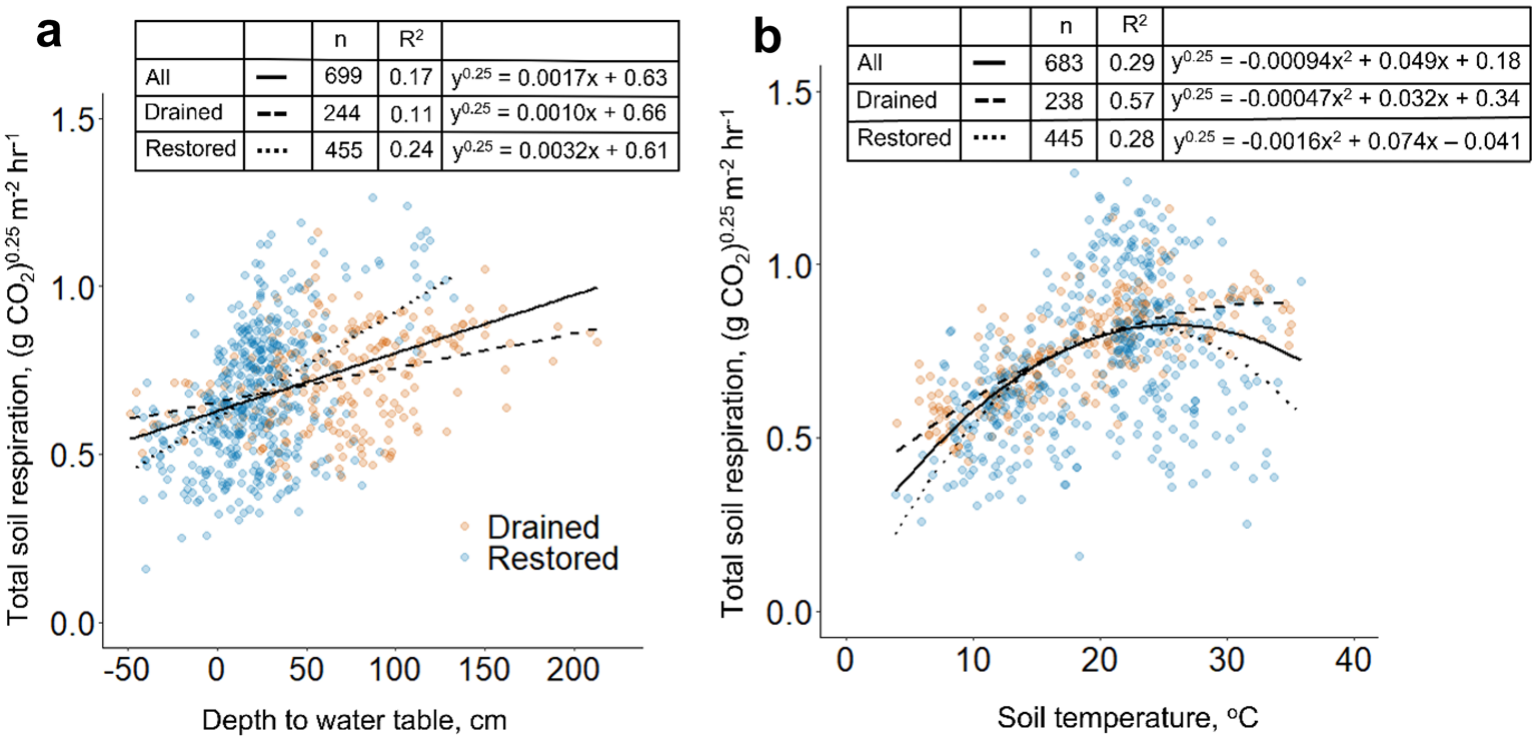
Power-transformed total soil respiration as a function of water table (**a**) and soil temperature (**b**) measured in drained and restored peatlands. Total soil respiration, depth to water table, and soil temperature were measured in drained and restored peatlands at four locations in eastern North Carolina and southeastern Virginia from 2007 to 2017. All coefficients are significant at P < 0.0001

Relationships among total soil respiration, depth to water table, and soil temperature are presented in Fig. 2. In drained and restored peatlands, total soil respiration increased as depth to water table increased (Fig. 2a). Total soil respiration increased with increasing soil temperature, peaking at 25 °C and decreasing at higher temperatures (Fig. 2b). Total soil respiration was more tightly linked to water table depth in restored than drained peatlands (Fig. 2a) while the opposite was true for soil temperature (Fig. 2b). The temperature sensitivity (Q10) of total soil respiration tended to increase with increasing average annual depth to water table (Additional file 1: Fig. S2).

The relationship between total soil respiration and depth to water table level was functionally different for drained and restored peatlands (ANCOVA, p = 0.002). Likewise, drainage status was a significant factor in the model relating total soil respiration to soil temperature (ANCOVA, p < 0.0001). Location was a significant factor in both models, indicating that the relationships among total soil respiration, depth to water table, and soil temperature differed among locations (ANCOVA, p < 0.0001 for both models).

When drained and restored peatlands were considered collectively, variation in soil respiration was not well explained by depth to water table (Fig. 2a) or soil temperature (Fig. 2b) alone. Together water table level and soil temperature explained 41% of the variation in power-transformed total soil respiration (p < 0.0001, n = 573) across drained and restored peatlands. Variance inflation factors (VIF) were equal to 1.0 for both independent variables in the multiple regression model.

## Discussion

### Linking total soil respiration to water table depth and soil temperature

Total soil respiration increased with increasing depth to water table and increasing temperature in drained and restored peatlands at three locations in eastern North Carolina and one location in southeastern Virginia, in agreement with previous studies in the region (34, 47—48). The strength of the relationship between soil respiration and soil temperature was enhanced in drained peatlands where soil temperature explained more variation in soil respiration than water table level (Fig. 2). This result, together with a trend towards rising temperature sensitivity of total soil respiration to soil temperature, or Q10, with increasing annual average water table depth (Additional file 1: Fig. S2), suggests that increasing drainage intensity amplifies peatland vulnerability to warming temperatures. This is an important finding of this meta-analysis that spanned a broad range of sites, implying exponential growth in future CO_2_ emissions from drained peatlands with global warming projections [49]. On the other hand, conservation of intact peatlands and restoration of drained peatlands may protect peatland soil carbon stocks from warming temperatures [50], offering climate benefits by avoiding increased CO_2_ emissions from peatlands in the future.

A reduction in depth to water table, as occurred during hydrologic restoration, increases water-filled pore space throughout the soil profile, imposing oxygen constraints on aerobic microbial respiration [51]. Indeed, depth to water table was a more important control on total soil respiration than soil temperature in restored peatlands (Fig. 2a, b), with soil respiration responding more strongly to changes in depth to water table in restored than drained peatland. SOM quality influences peat decomposition rate (52–54), and drained peats may have had a higher ratio of recalcitrant to labile carbon compounds than restored peats due to advanced peat decomposition after many years of drainage (Table 1), making them less sensitive to changes in depth to water table. Fires, which are more frequent in drained peatlands, also degrade SOM quality, creating recalcitrant “black carbon” at the soil surface [55, 56]. Wetland conversion and restoration also alters soil microbial communities [36, 37] which may influence the response of soil respiration to variation in physical drivers [57].

While direct comparison of restored sites to equivalent drained sites in southeastern U.S. peatlands indicates that raising water table levels reduces total soil respiration rates [18, 19], our experimental design did not control for the impact of variation in environmental variables such as peat chemistry and vegetation among locations and sites and inter-annual variation in precipitation and temperature on soil respiration. While mean annual depth to water table was greater in drained than restored plots, mean annual total soil respiration and soil temperature in drained and restored plots were not significantly different (Table 4), indicating that other factors such as differences in vegetation (Table 1) and peat chemistry (Table 2), that differed among locations, influenced soil respiration in addition to depth to water table and soil temperature. Indeed, location was a significant factor in ANCOVA. Total soil respiration also tended to be higher overall at HF compared to other locations, which may have been related to hotter and drier climatic conditions during the measurement period at this site compared to other sites included in our analysis (Table 1). At HF, total soil respiration tended to be greater in the forested restored site than the drained site with herbaceous vegetation cover (Table 3). This was likely due to a larger contribution of autotrophic respiration from forest vegetation compared to herbaceous vegetation in drained plots, as soil respiration has been found to be correlated with leaf area index and aboveground litterfall in peat-forming ecosystems [58]. Declining heterotrophic respiration has been linked to decreases in peat C:N ratio in simulations of drained tropical peatlands [59] and low peat C:N ratio at the restored TLRP site may have driven low total soil respiration rates in addition to high water table levels. By aggregating data from different locations and measurement periods (Table 1), we sampled a range of environmental conditions, thereby capturing the influence of a wide range of climatic conditions and variation in SOM substrate quality and vegetation on rates of peat decomposition.

### Estimating CO_2_ emissions from peat decomposition

Raising water table levels in drained peatlands of the southeastern United States has been identified as an important mechanism for reducing anthropogenic CO_2_ emissions [16]. Carbon offset markets can provide partial financing for hydrological restoration, but robust methods to estimate net GHG impacts are needed to quantify the atmospheric benefit of restoration to justify funding. Using models presented here, researchers and managers can estimate total soil respiration based on parameters that are easily measured and monitored in the field, partition model outputs to estimate the contribution of heterotrophic respiration from peat decomposition, and thereby contemplate potential climate change mitigation benefits of peatland hydrological restoration without having to undertake complex GHG flux assessments.

Complete peat CO_2_ budgets considering all sources of C inputs (litterfall and root mortality) and outputs (heterotrophic respiration and lateral carbon transport) are needed to assess the net impact of peatland drainage and hydrological restoration on peat CO_2_ emissions [60]. In addition, peatland drainage and restoration impact non-CO_2_ emissions (CH_4_, N_2_O) [8, 24, 61] as well as C storage in aboveground and belowground vegetation [62]. Our results also do not account for GHG emissions from CH_4_ or N_2_O, two potent greenhouse gases that are produced under anaerobic or transitional conditions in peatland soils [61, 63]. Further research can help to determine GHG emissions from CH_4_ and N_2_O as well as the influence of fluctuating C inputs to peat soil from litterfall and root mortality and C leaching. Nonetheless, empirical models relating soil respiration rates to environmental drivers could help to significantly decrease costs of quantifying the benefits of peatland restoration on CO_2_ emissions from peat decomposition, because direct measurements of soil respiration are time consuming and expensive. For example, according to the model relating total soil respiration to water table level presented here (Fig. 2a), extrapolating hourly fluxes to a full year, and applying a global average partitioning ratio to estimate the contribution of heterotrophic respiration (50%, 64), raising the mean annual depth to water table from 60 to 10 cm over an area of 500 ha would reduce CO_2_ emissions from peat soil by roughly 5000 Mg CO_2_e over a period of two years. At a price of 5 USD per Mg on the voluntary market [65], and accounting for buffer contributions to mitigate non-permanence risk, these credits could generate around 25,000 USD to fund restoration. The cost of measuring CO_2_ emissions at P-D-2 and P-R-2 for two years was approximately 90,000 USD (including personnel costs, travel to field sites, static chamber construction, and gas sample analyses). Equipment that can measure water table level and soil temperature can now be purchased for approximately 3000 USD. Though there will still be personnel costs associated with field deployment and data downloads, this represents a substantial cost reduction for land managers interested in using proxy measurements to estimate reductions in CO_2_ emissions from peat soil from peatland restoration.

With Mean Bias Error (MBE) < 0, the water table, soil temperature, and combined models all underestimated total soil respiration at P-R-2 (Additional file 1: Fig. S1), a site that had active restoration and relatively high rates of total soil respiration among the sites (Table 3). Nonetheless, large-scale application of the model across geographic locations, land use histories, and drainage would generate a largely unbiased estimate of reductions in CO_2_ emissions resulting from hydrological restoration (Additional file 1: Fig. S1). Our power-transformed model notably underestimated total soil respiration when observed values were larger than 1 g CO_2_ m^−1^ h^−1^ (Additional file 1: Fig. S1). However, in our dataset representing a decade of peatland measurements across two states, only 7.4% of total soil respiration observations were greater than 1 g CO_2_ m^−1^ h^−1^ and only 2.4% were greater than 1.5 g CO_2_ m^−1^ h^−1^. Therefore, our model performs adequately in 92.6% of conditions measured regionally over multiple years. In addition to functional differences in the relationships among total soil respiration, depth to water table, and soil temperature in drained and restored peatlands, differences amongst locations influenced variation in the response of soil respiration to physical drivers. Further refinement of the models presented in this study could reduce uncertainty in estimates of reduced CO_2_ emissions from peat decomposition resulting from hydrological restoration of drained peatlands. In particular, increased understanding of the influence of changes in peat chemistry and fluctuations in soil moisture in surface layers in drained and restored peatlands could improve model accuracy. Additional areas for improvement include studies that partition soil respiration into heterotrophic and autotrophic components in peatlands of the southeastern U.S. coastal plain as well as measurements of all peat C inputs and outputs to generate full net peat CO_2_ budgets.

## Conclusions

Peatland restoration can contribute to nature-based solutions to mitigate climate change, while providing other benefits such as wildlife habitat, flood protection, and water quality improvements and catastrophic wildfire risk reduction. Our results suggest that drained peatlands in the southeastern United States are more vulnerable to warming temperatures than hydrologically restored peatlands. Applying models developed in this study with partitioning ratios to estimate the heterotrophic contribution to total soil respiration, water table level and soil temperature can be monitored to estimate the reductions in CO_2_ emissions from peat decomposition generated by hydrological restoration. Additional research on drivers of heterotrophic respiration in peatlands across the southeastern U.S. coastal plain could further reduce uncertainty in emissions from drained peatlands and the potential reduction in CO_2_ emissions generated by peatland restoration. Full accounting of GHG benefits, however, includes all emissions, sources, and sinks along with CO_2_ emissions from heterotrophic respiration. Growth in carbon offset markets could increase funding available for peatland restoration, and accurate estimates of GHG emission reductions resulting from raising water table levels in drained peatlands are important components of these initiatives.

## Supplementary Information

**Additional file 1: Figure S1.** Total soil respiration predicted by depth to water table (CO2, g m-2 hr-1 ^0.25, = -0.0017 * depth to water table, cm + 0.63) (a), soil temperature (CO2, g m-2 hr-1 ^0.25, = -0.00094 * soil temperature, oC^2 + 0.049 * soil temperature, oC + 0.18) (b) and combined water table level and soil temperature (CO2, g m-2 hr-1 ^0.25 = 0.0016 * depth to water table, cm - 0.00077 * soil temperature, oC^2 + 0.040 * soil temperature, oC + 0.21) (c) versus observed values of total soil respiration. Black circles: Testing data withheld from model development (n = 10); Grey crosses: full dataset for model development. **Figure S2.** Q10 and mean annual depth to water table in drained and restored peatlands in the southeastern United States.

## Data Availability

The dataset supporting the conclusions of this article is available in the Harvard Dataverse repository, https://dataverse.harvard.edu/dataset.xhtml?persistentId=doi:10.7910/DVN/QSMOI8
