# Supplementary material for: Response of soil respiration to changes in soil temperature and water table level in drained and restored peatlands of the southeastern United States
Source: Carbon Balance Manag. 2022 Nov 19;17:18. doi: 10.1186/s13021-022-00219-5 (PMC9675111; doi:10.1186/s13021-022-00219-5)
Supplement: Supplementary file 1 — Additional file 1: Figure S1. Total soil respiration predicted by depth to water table (CO2, g m-2 hr-1 ^0.25, = -0.0017 * depth to water table, cm + 0.63) (a), soil temperature (CO2, g m-2 hr-1 ^0.25, = -0.00094 * soil temperature, oC^2 + 0.049 * soil temperature, oC + 0.18) (b) and combined water table level and soil temperature (CO2, g m-2 hr-1 ^0.25 = 0.0016 * depth to water table, cm - 0.00077 * soil temperature, oC^2 + 0.040 * soil temperature, oC + 0.21) (c) versus observed values of total soil respiration. Black circles: Testing data withheld from model development (n = 10); Grey crosses: full dataset for model development. Figure S2. Q10 and mean annual depth to water table in drained and restored peatlands in the southeastern United States. [file 13021_2022_219_MOESM1_ESM.docx]

Additional Information

**Response of soil respiration to changes in soil temperature and water table level in drained and restored peatlands of the southeastern United States**

*S1. Supplementary information on study sites*

Study sites were comprised of drained and restored pocosin peatlands. Although the term pocosin has also been applied broadly to all shrub and forested bogs, as well as Atlantic white cedar stands and some loblolly pine stands on flooded soils of the southeastern coastal plain of the United States (Wells, 1928; Woodwell, 1958; Kologiski, 1977), here we use the term pocosin to refer to freshwater wetlands, with some component of broad-leaved evergreen shrubs or low trees, on organic soils in the coastal plain of southeast Virginia, North Carolina, South Carolina, or Georgia. Much of this landscape was logged in the 19^th^ and 20^th^ centuries; large-scale conversion of pocosins to agriculture occurred in the 1970 – 1980s with the construction of large canals and drainage systems (Carter, 1975).

The Great Dismal Swamp (GDS) encompasses over 54,000 ha of freshwater forested wetlands located in southeastern Virginia and northeastern North Carolina. Prior to European settlement, GDS included wetland forests dominated by bald cypress (*Taxodium distichum*) and Atlantic white cedar (*Chamaecyparis thyoides*) (Barrd 2006) extending over an estimated area of 404,000 ha (Laderman et al., 1989; Oaks & Whitehead, 1979). Beginning in the mid-1700s and continuing for approximately 200 years, these forests were drained, cultivated, and logged (Lilly, 1981), reducing them to their current extent. Commercial logging and drainage were discontinued in 1974 when the GDS became part of the U.S. Fish and Wildlife National Wildlife Refuge system. However, drained conditions persist at GDS due to roughly 250 km of remaining ditches and roads (Barrd 2006). Remnant populations of Atlantic white cedar and baldcypress are present along with pond pine (*Pinus serotina*), but red maple (*Acer rubrum*), black gum (*Nyssa sylvatica*), and sweet bay (*Liquidambar syraciflua*) have become a major part of the forest composition (Laderman et al., 1989). Measurements presented in this study were collected from 2015 – 2017 in maple-gum, pine-shrub, and cedar forest types (Gutenberg et al., 2019). GDS includes both rain-fed ombrotrophic bogs which depend on precipitation for nutrient inputs as well as minerotrophic fens receiving nutrient inputs from groundwater flow (Drexler et al., 2017). Peat depths at the site range between 2 and 3 m (Oaks & Whitehead, 1979).

Hofmann Forest (HF) lies on what historically has been known as the White Oak pocosin (Daniels et al., 1977). Measurements were collected from 2011 – 2012 at HF in a fallow field where no crop had grown since 2009 and in a previously drained and converted wetland area that was restored in 2005 (O’Doherty, 2013). The vegetation in the fallow field was mostly herbaceous and dominated by horseweed (*Conyza canadensis*). The restored wetland was planted in a variety of tree species, including longleaf pine (*Pinus palustris*), bald cypress (*Taxodium distichum*), and sawtooth oak (*Quercus acussitima*). The understory of the restored wetland consisted mainly of blueberry (*Vaccinium sp.*), greenbriar (*Smilax sp.*), and grasses.

Pocosin Lakes National Wildlife Refuge (PLNWR) was established on peatlands previously drained for agriculture. Beginning in the mid-1990s, refuge management began gradually blocking drainage canals to restore pre-drainage water levels. Measurements presented in this study were collected from 2011 – 2013 and 2016 – 2017. The 2011 – 2013 measurements were carried out in an area of the refuge that remained drained and in a site restored 20 years previously when the refuge was created (Wang et al., 2015). Measurements collected from 2016 – 2017 were implemented in the same drained site within the refuge and one additional drained site. At the drained site measured 2011 – 2013 and 2016 – 2017, the water tables were raised in March 2017 using water control structures to regulate the release of water from the area. The dominant species in restored areas at PLNWR are inkberry *(Ilex glabra*), large gallberry (*Ilex coriacea*), honeycup (*Zenobia pulverulenta*), fetterbush (*Lyonia lucida*) and laurel greenbrier (*Similax laurifolia*) with some smaller pond pine (*Pinus serotina*) and loblolly pine (*Pinus taeda*) trees (Armstrong et al., in press; Wang et al., 2015). Western braken fern (*Pteridium aquilinum*) is the predominant groundcover in drained areas with scattered winged sumac (*Rhus copallinum*), wax myrtle (*Morella cerifera*) and titi (*Cyrilla racemiflora*) shrubs. Peat depths in the refuge reach up to 3 m (Armstrong et al., in press; Wang et al., 2015).

Swamp forests at the Timber Lakes Restoration Project (TLRP) site were partially cleared, drained, and converted to agriculture in the 1970s. Measurements presented in this study were collected from a former corn and soybean farmland within TLRP that was restored in 2004 (Morse et al., 2012). Restoration of the area was achieved by leveling the area and filling drainage ditches followed by tree planting. Obligate and facultative wetland tree species were planted, including bald cypress (*Taxodium distichum*)*,* black gum, (*Nyssa sylvatica*), water tupelo (*Nyssa aquatica*), green ash (*Fraxinus pennsylvanica*), black willow (*Salix nigra*), Atlantic white cedar (*Chamaecyparis thyoides*), water oak (*Quercus nigra*), swamp chestnut oak (*Quercus michauxii*), willow oak, (*Quercus phellos)*, and cherrybark oak (*Quercus falcata var pagodifolia*) (Needham, 2006). The TLRP property drains to the Little Alligator River, historically forming the source of coastal blackwater streams (Needham, 2006). Upstream and downstream hydrological connections were restored to the area in 2007 (Ardón et al., 2010). Peat depth at the site was at least 15 cm (Morse et al., 2012).

*S2. Supplementary information on model testing*

*S2.1 Statistics*

Using the models generated by the regression analysis for combined drained and restored peatlands and the withheld data, we computed model error as the Root Mean Square Error (RMSE) and Mean Bias Error (MBE) (Hardel & Simar, 2007). We additionally assessed association between predicted and observed values using R^2^ (Smith et al., 1996). To further assess uncertainty in total soil respiration values predicted by models considering drained and restored peatlands together, we calculated the half width of the 90% confidence interval as a percentage of the predicted value.

*S2.2 Results*

Back-transformed predicted values are compared to observed values in Figure S1. With RMSE of 0.18 g CO_2_ m^-2^ hr^-1^, the model using water table level to predict total soil respiration generated values closer to observed total soil respiration than models using soil temperature (RMSE = 0.22 g CO_2_ m^-2^ hr^-1^) or the combined model (RMSE = 0.22 g CO_2_ m^-2^ hr^-1^) (n=10, Figure S1). The MBE of the water table, soil temperature, and combined model was -0.07, -0.10, and -0.02 g CO_2_ m^-2^ hr^-1^ respectively.


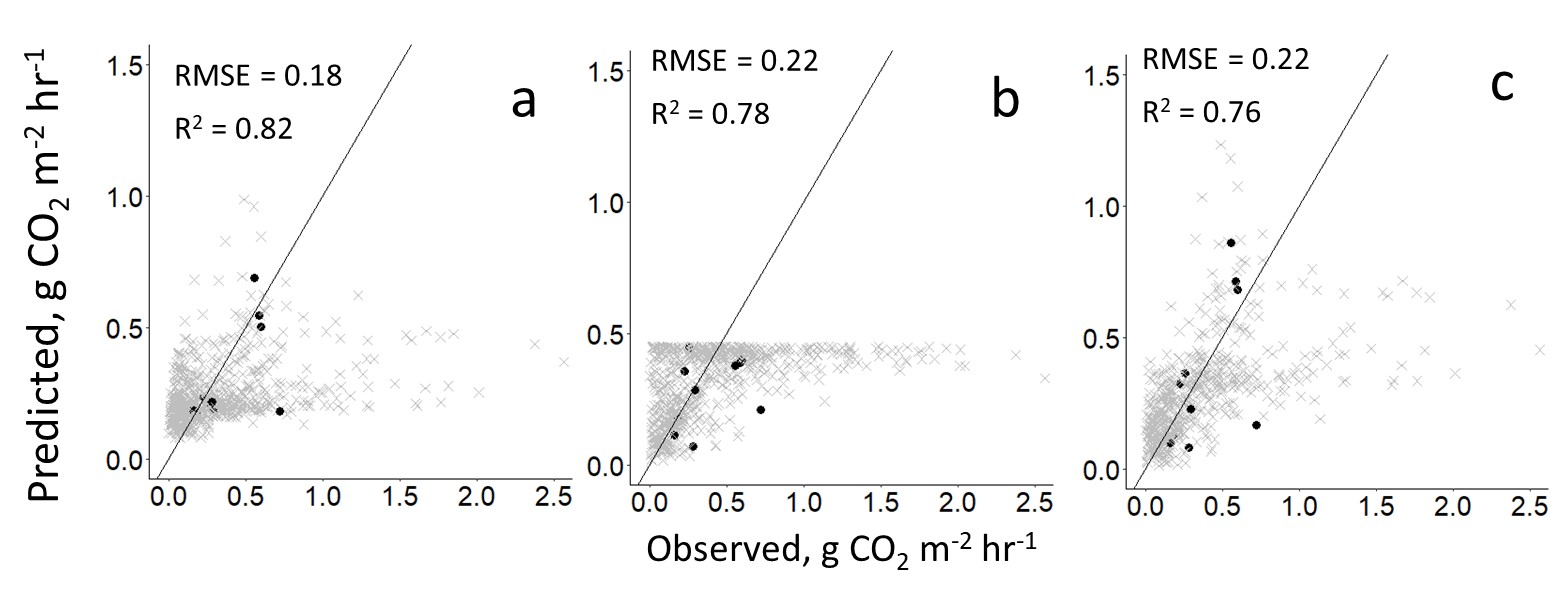


**Figure S1. Total soil respiration predicted by depth to water table (CO_2,_ g m^-2^ hr^-1^ ^0.25, = -0.0017 * depth to water table, cm + 0.63) (a), soil temperature (CO_2,_ g m^-2^ hr^-1^ ^0.25, = -0.00094 * soil temperature, ^o^C^2 + 0.049 * soil temperature, ^o^C + 0.18) (b) and combined water table level and soil temperature (CO_2,_ g m^-2^ hr^-1^ ^0.25 = 0.0016 * depth to water table, cm - 0.00077 * soil temperature, ^o^C^2 + 0.040 * soil temperature, ^o^C + 0.21) (c) versus observed values of total soil respiration.** Black circles: Testing data withheld from model development (n = 10); Grey crosses: full dataset for model development.

The half width of the 90% confidence interval of the regression equation relating soil respiration to water table (CO_2,_ g m^-2^ hr^-1^ ^0.25, = -0.0017 * depth to water table, cm + 0.63) was less than 10% of the predicted value of total soil respiration at water table level values from 117 cm below the soil surface to 13 cm above the soil surface. The half width of the 90% confidence interval of the regression equation relating soil respiration to soil temperature (CO_2,_ g m^-2^ hr^-1^ ^0.25, = -0.00094 * soil temperature, ^o^C^2 + 0.049 * soil temperature, ^o^C + 0.18) was less than 10% of the predicted value of total soil respiration at soil temperature values from 11 ^o^C to 33 ^o^C. The half width of the 90% confidence interval of the multiple regression equation (CO_2,_ g m^-2^ hr^-1^ ^0.25 = 0.0016 * depth to water table, cm - 0.00077 * soil temperature, ^o^C^2 + 0.040 * soil temperature, ^o^C + 0.21) was less than 10% of the predicted value of total soil respiration over the two-dimensional space of predictor variables defined by depth to water table and soil temperature values of 95 cm, 30 ^o^C; 95 cm, 15 ^o^C; -10 cm, 15 ^o^C; -10 cm, 25 ^o^C, with negative depth to water table values indicating water table level above the soil surface.

*S3. Supplementary information on temperature sensitivity of total soil respiration*

To further explore the temperature sensitivity of total soil respiration, we computed for each site a Q10 value that is the factor by which soil respiration increases for a 10 ^o^C increase in temperature (e.g., Kirschbaum, 1995, Van’t Hoff, 1898). We fit an exponential equation to total soil respiration and temperature at each site using monthly measurements (Equation 1):

SR = a * e^b*T^ (Eq 1)

Where SR: Total soil respiration, Mg CO_2_ ha^-1^ yr^-1^; T: Soil temperature, ^o^C. The variables a and b are fitted parameters.

The Q10 value was then calculated by inserting the parameter b into Equation 2:

Q10 = e^b*10^ (Eq 2)

Q10 values could only be calculated for eight of the ten sites since soil temperature measurements were not available for PLNWR1 or PLNWR2. The Q10 values ranged from 1.3 to 4.1. To investigate relationship between total soil respiration temperature sensitivity and water table level, we regressed Q10 values against the mean annual water table level at each site. Q10 tended to decrease with increasing annual mean water table level (Figure S2).


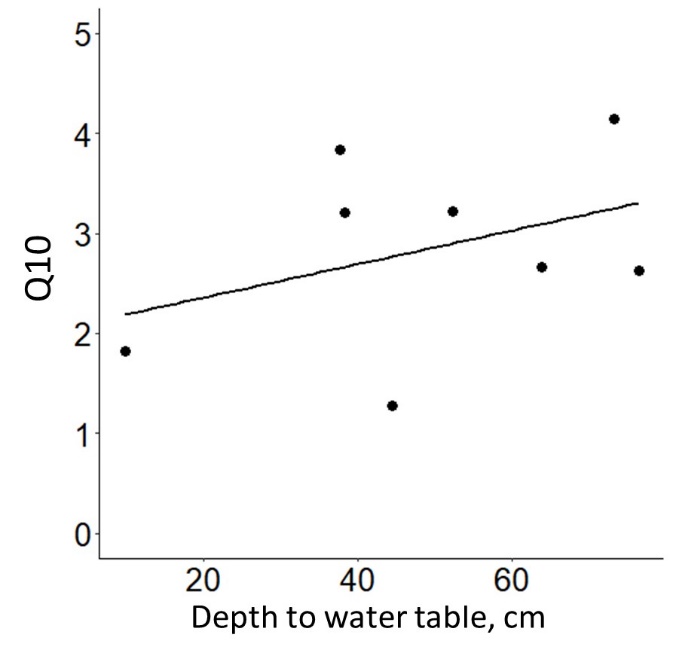


**Figure S2. Q10 and mean annual depth to water table in drained and restored peatlands in the southeastern United States.**

*S4. References of supplementary material*

Ardón, M., Morse, J.L., Doyle, M.W. and Bernhardt, E.S. (2010). The water quality consequences of restoring wetland hydrology to a large agricultural watershed in the southeastern coastal plain. *Ecosystems*, *13*(7), 1060-1078.

Armstrong, L., Peralta, A., Krauss, K.W., Cormier, N., Moss, R.F., Soderholm, E., McCall, A., Pickens, C., Ardón, M. Hydrologic restoration decreases greenhouse gas emission from shrub bog peatlands in Southeastern U.S. In review.

Barrd, S.C. (2006). Great Dismal Swamp National Wildlife Refuge and Nansemond National Wildlife Refuge Final Comprehensive Conservation Plan July 2006. U.S. Fish and Wildlife Service Comprehensive Conservation Plan.

Carter, L.J. (1975). Agriculture: A new Frontier in Coastal North Carolina. *Science,* *189*(4199), 271-275.

Daniels, R. B., Gamble, E. E., Wheeler, W. H., Holzhey, C. S. (1977). The stratigraphy and geomorphology of the Hofmann Forest Pocosin. *Soil Science Society of America Journal, 41*(6),1175-1180.

Drexler, J.Z., Fuller, C.C., Orlando, J., Salas, A., Wuster, F.C., Duberstein, J.A. (2017). Estimation and uncertainty of recent carbon accumulation and vertical accretion in drained and undrained forested peatlands of the southeastern USA. *Journal of Geophysical Research: Biogeosciences, 122*(10), 2563-2579.

Gutenberg, L., Krauss, K., Qu, J., Ahn, C., Hogan, D., Zhu, Z., Xu, C. (2019). Carbon dioxide emissions and methane flux from forested wetland soils of the Great Dismal Swamp, USA. *Environmental Management,* *64*(2), 190-200.

Kirschbaum, M. (1995). The temperature dependence of soil organic matter decomposition, and the effect of global warming on soil organic C storage. *Soil Biology and Biochemistry, 27*(6), 753–760.

Kologiski, R.L. (1977). The phytosociology of the Green Swamp, North Carolina. North Carolina Agricultural Experiment Station, Raleigh, N.C., Technical Bulletin No. 250, 101 pp.

Laderman, A.D., Brody, M., Pendleton, E. (1989). The ecology of Atlantic white cedar wetlands- a community profile. U.S. Department of Interior, Fish and Wildlife Service, National Wetlands Research Center. Biological Report 85(7.21), 114 pp.

Lilly, J. P. (1981). A history of swamp land development in North Carolina. In Proceedings of Pocosins: A Conference on Alternative Use of the Coastal Plain Freshwater Wetlands of North Carolina (pp. 20–39). Beaufort, NC: Duke University Marine Laboratory.

Morse, J., Ardón, M., Bernhardt, E. (2012). Greenhouse gas fluxes in southeastern U.S. coastal plain wetlands under contrasting land uses. *Ecological Applications,* *22*(1), 264-280.

Needham, R. (2006). Implementation plan for agricultural restoration at Timberlake Farms. Needham Environmental, Wilmington, North Carolina, University.

Oaks, R.Q., Whitehead, D.R. (1979). Geologic setting and origin of the Dismal Swamp, southeastern Virginia and northeastern North Carolina. In: P.W. Kirk PW (Ed.), *The Great Dismal Swamp* (pp. 1-24). University Press of Virginia, Charlottesville.

O’Doherty, C. (2013). An assessment of soil carbon dioxide respiration and environmental influences for undisturbed, drained and restored wetlands. Masters thesis. University of North Carolina, Raleigh, North Carolina, USA.

Van’t Hoff, J. H. (1898). Lectures on theoretical and physical chemistry. In Chemical Dynamics Part I (pp. 224–229). London: Edward Arnold.

Wang, H., Richardson, C.J., Ho, M. (2015). Dual controls on carbon loss during drought in peatlands. *Nature Climate Change, 5*(6), 584-587.

Wells, B.W. (1928). Plant communities of the coastal plain of North Carolina and their successional relations. *Ecology 9*(2), 230-242.

Woodwell, GM. (1958). Factors controlling growth of pond pine seedlings in organic soils of the Carolinas. *Ecological Monographs* *28*(3), 219–236.
